# Barriers to and Facilitators of Community Participation and Empowerment in Primary Health Care Centres in Italy: The Perspectives of Stakeholders and Practitioners

**DOI:** 10.3390/healthcare14142050

**Published:** 2026-07-08

**Authors:** Daniela Rosalba Luisi, Kerstin Hämel

**Affiliations:** 1 Department of Health Services Research and Nursing Science, School of Public Health, Bielefeld University, 33615 Bielefeld, Germany; daniela.luisi@uni-bielefeld.de; 2 Department of Business Economics, Health and Social Care, University of Applied Sciences and Arts of Southern Switzerland (SUPSI), CH-6928 Manno, Switzerland; 3 Department of Nursing Science, Faculty of Social Sciences, University of Vienna, 1090 Vienna, Austria

**Keywords:** Case della Salute, Case della Comunità, primary care, involvement, engagement, community health centres, qualitative research

## Abstract

Background/Objectives: Community participation and community empowerment are key features of people-centred primary health care systems and are crucial ways to make health services more equitable and appropriate for people’s needs while improving these services. This qualitative study investigates the facilitators and barriers that practitioners and stakeholders encounter in their efforts to support community participation and community empowerment in primary health care centres. In this context, we focus on the Case della Salute/Case della Comunità in Emilia-Romagna, Italy. Methods: We conducted semistructured interviews with 19 practitioners and stakeholders who had practical or research experience with this topic from October 2020 to May 2021. These interviews were analysed inductively via qualitative content analysis. Results: The barriers and facilitators identified in this context were related to four themes: (a) the culture and mindset of practitioners and organisations as well as the competences that enable them to support community participation and empowerment; (b) reframing local health care politics, the organisation and the role played by health care organisations in this context; (c) investing human and financial resources and creating appropriate spaces for community participation and empowerment; and (d) operational aspects that facilitate community participation and empowerment. Conclusions: The study presents various barriers and facilitators that operate at different levels, such as the level of the health care system, the organisational level and the operational level. We discuss the barriers and facilitators that are encountered in this context and offer suggestions regarding what stakeholders, managers and practitioners can learn from these insights.

## 1. Introduction

Countries worldwide have been called upon to strengthen their primary health care (PHC) systems to meet both present and future demographic and epidemiological challenges [1,2,3]. Strong PHC systems have been linked to improved access to integrated, person-centred care and better continuity of care [3,4,5]. PHC has been defined as a “whole-of-society approach to health that aims to maximise the level and equitable distribution of health and well-being” [4] (p. 4). This goal can be achieved by prioritising people’s needs and opinions in all areas, ranging from health promotion and prevention to treatment, rehabilitation and palliative care [4]. The development of strong PHC systems has been identified as an important strategy for making health care systems and organisations more people centred [6].

The fundamental features of people-centred PHC systems are community participation (CP) and community empowerment (CE); these approaches focus on involving people in decision-making processes that pertain to their own life situations [4]. CE is defined as an approach that provides opportunities for people and communities to make decisions and take control over and improve their own health and life through the development of knowledge, skills, power and resources. In this context, CP refers to communities’ engagement in decision-making and the design, implementation and evaluation of actions related to health and well-being [6].

The World Health Organisation’s (WHO) Framework on Integrated People-Centred Health Services explicitly identifies “engaging and empowering people and communities” as an important strategy to make health services more appropriate for people’s needs [6]. Approaches rooted in the concepts of CP and CE have the potential to improve health services and health outcomes, increase the efficiency of care and promote equity and social justice [7,8].

In 2006, Italy introduced the community health centre (CHC) model known as “Case della Salute” (“Houses of Health”) to strengthen its PHC system [9,10,11]. The Emilia-Romagna region was one of the first Italian regions to implement this model. From 2010 on, this region adopted various policies to strengthen the health centre’s community-oriented approach by stating that CP and CE are fundamental principles of the CHCs and must be promoted [9].

In 2022, with Italy’s important PHC reform through Ministerial Decree 77/2022 [12], CHCs were renamed “Case di Comunità o Case della Comunità” (“Houses of the Community”) with the aim of emphasising the importance of a community-oriented PHC model [11,12]. These multidisciplinary health care facilities are part of the broader first-level PHC system and exist in parallel to general practitioners’ single practices and other PHC services. With the new PHC reform, CHCs now play a central role in providing comprehensive PHC [12]. Compared with traditional primary care practices, the added value of CHCs is found in their interprofessional work, the integration of health and social services, a population-oriented and territorial approach to health, and the support of CP and CE within a given territory [12,13,14]. In Italy, CHCs are organised on the basis of a hub and spoke model and are managed by local health care trusts (LHCTs) [12].

Moreover, Decree 77/2022 strengthened the role of the health districts which are local organisational and functional branches of the LHCT within a given territory and responsible for a defined population. With the reform they shift from administrative units to strategic organisers of community care: Their tasks now encompass district social and health needs assessment, budgeting, planning and delivery of territorial health services, coordination across primary care and domiciliary care, and the integration of social and health services, also through the CHCs [12].

Table 1 presents an overview of the number of inhabitants served as well as the professionals who practice in the health centres.

Italian CHCs are responsible not only for health and social services but also for promoting CP and collaborating with citizens, service users and volunteer associations [9,12]. In accordance with the Ministerial Decree of 2022 [12] and the first national guidelines on CP and CE development in CHCs [16], health centres in all Italian regions are currently obligated to develop CP through processes that involve coplanning and codesigning, particularly in the fields of health promotion, prevention and care [12,15,16].

The transformation of PHC systems, as in the case of Italy, requires changes at different levels of the health care system [3]. As described by the WHO [3], the introduction of this type of innovation requires clear argumentation to ensure sufficient legitimacy for change, sustained leadership from both political actors and management, flexibility in terms of organisational design and appropriate resource allocation. Moreover, the participation and empowerment of communities in PHC is crucial for the shift towards PHC models that seek to promote health and well-being and focus on social determinants of health. Accordingly, the introduction of CHCs requires changes at the organisational and management levels as well as at the levels of practitioners, users and communities.

In a previous document analysis study of regional Emilia-Romagna and national Italian policies, we explored the over-time evolution of the conceptualisation of CP and CE in relation to Italian CHCs in policy formulation. The results of this study indicate that although the policies strengthen the idea of democratic practice and shared decision-making power with communities, clear strategies for how to involve disadvantaged communities in CP processes and how to support CE are lacking. Moreover, there is little discussion of how third-sector organisations that have an ambiguous role in this PHC context (i.e., the role of service providers and representatives of the community) can be adequately involved [9].

In a second study, we explored the understanding of CP and CE among practitioners and stakeholders who design participatory approaches in the Italian CHCs. The results suggest that CP and CE are defined as a variety of forms of dialogue and collaboration, such as listening to people’s needs during public consultations or cooperation with community subgroups, such as pupils or seniors, through formal and informal networks. Moreover, CP and CE are often used as means for the development of services and to improve service quality, e.g., to better address community health needs. Our results highlight CP and CE as levers for collectivism, empowerment, and democracy in health and as stimuli for changes in health institutions; that is, CP and CE function as catalysts for practitioners to develop skills and new community working methods [17].

However, only a few studies in the context of high-income European countries have identified barriers and facilitators related to the development of CP and CE in PHC (e.g., [18,19,20,21]). Moreover, while different CP and CE approaches, such as CP in governance structures (e.g., in CHC boards) or the participation of community groups in specific activities and projects, are recognised as important for European health centres, their development in practice is considered challenging [14].

Given this background, a comprehensive analysis of the facilitators of and barriers to CP and CE support in European CHCs, such as Italian CHCs, and a discussion of policy and practice implications are needed. This is particularly relevant for the renewed Italian CHC context, which is understudied in this regard.

### Aim of the Study

The aim of this study is to explore the barriers to and facilitators of CP and CE promotion in CHCs in Emilia Romagna, Italy, from the perspectives of practitioners and stakeholders.

The focus on practitioners’ and stakeholders’ perspectives provides valuable insights into not only microlevel CP and CE development but also behind-the-scenes changes at the meso- and macrolevels. On the basis of practitioners’ experiences of good-practice examples drawn from CHCs and practitioners’ and stakeholders’ expertise at the meso- and macrolevels, we aim to provide a detailed and comprehensive view of the topic.

## 2. Materials and Methods

### 2.1. Study Design

We employed a qualitative study design with semistructured interviews and analysed them using inductive qualitative content analysis adapted from Kuckartz & Rädicker [22]. The in-depth analysis focused on stakeholders’ and professionals’ views on facilitators of and barriers to promoting CP and CE processes [23]. The interview partners included regional and local practitioners from the Emilia-Romagna region and local, regional and national stakeholders who possessed specialised knowledge of the processes and contexts associated with CP and CE in the field of PHC [24].

In a previous analysis of these interview data, we explored the conceptualisations of, and meanings attributed to CP and CE by the practitioners and stakeholders [17]. In the current study, we were particularly interested in their opinions and experiences of the barriers and facilitators they encounter when aiming to support the community through participatory processes.

A third analysis of the data, which will be published later, addresses the questions of which methods are used in practice to promote CP and CE, which communities are targeted, which goals are addressed, and what benefits are expected and/or experienced. Moreover, the third analysis will provide specific examples of CP and CE projects and categorise them according to the levels of participation [25]. Sampling, field access and data collection for the interview study were described in detail elsewhere [17]. All three explorations address different research questions and use distinct analyses. For the current study, the main methodological steps involved a strong inductive approach and the construction of a specific coding system. In contrast, in the other two analyses of the interview material, we applied two distinct analysis methods: a deductive–inductive content analysis approach [17] and a deductive approach in which the data were strongly guided by the levels of participation [25].

### 2.2. Sampling and Field Access

Our sample consisted of practitioners and stakeholders who had different professional backgrounds and were involved in the promotion of CP and CE in the PHC context. We defined the term “practitioners” in this context to include all individuals who worked in or collaborated with CHCs and who were involved in the process of promoting CP and CE processes at the operational level. Stakeholders included informants at the local, regional and national levels (such as local managers, researchers and representatives of associations in the third sector operating in the field of PHC) who had conducted research or employed related processes and had thus obtained a more comprehensive perspective on this topic. In the context of Italian CHCs, the third sector includes a variety of non-profit organisations ranging from volunteer groups, patient and citizen organisations performing advocacy and citizens’ rights protection to organisations providing services [16].

To select participants, we followed a purposive and snowball sampling approach suggested by Patton [26] (see also [17]).

First, we recruited stakeholders who had comprehensive information about CP and CE in relation to CHCs and had obtained a good overview of good-practice examples in the region. On the basis of a literature analysis, PHC networks, websites of national and local governments, and policy documents, we were able to select relevant stakeholders. We contacted these stakeholders by e-mail and invited them to participate in an interview. Second, we identified good-practice examples of CHCs seeking to strengthen CP and CE on the basis of the recommendations of these stakeholders as well as through an analysis of the relevant literature and (policy) documents on this topic. We then contacted practitioners who were involved in CP and CE activities.

Given that CHCs have different dimensions and include different types of staff (e.g., hub/spoke CHCs), are located in diverse areas (rural/urban), operate under the auspices of differing local health care agencies and care for diverse populations, we introduced variation into the sample by including health centres from different provinces within the region that exhibited distinctive characteristics.

The recruitment process extended from the 2 October 2020 until the 31 May 2021. This was before the PHC reform of 2022, which further promoted CP and CE approaches by making them required parts of each CHC [12]. However, the idea of promoting CP was already part of the national CHC’s plans from the beginning of its introduction in 2006, and the Emilia-Romagna region demonstrated early investment in the development of CP and CE in these organisations [9]. We therefore draw on the solid experience of interview partners acting in the context of this region.

When the data we collected enabled us to provide full definitions of themes and the information that we received began to be repeated by the interview partners (i.e., when the point of data saturation had been reached), we stopped recruiting additional participants.

In total, 19 interview partners shared their experiences pertaining to 14 different CHCs. Among these participants, 13 were practitioners, such as staff members working in the health centres who demonstrated a coordination function with respect to CP and CE processes, as well as cooperating practitioners from local health care agencies or social services who were also involved in the activities in question (at the local level). Six interview partners were stakeholders, including representatives of associations from the third sector (the national level), researchers and project coordinators with a focus on CP and CE in the PHC context (the national and regional levels) and managers of the local PHC or health centres (the local level). Table 2 presents an overview of the characteristics of the sample.

### 2.3. Ethics

We received ethical approval to conduct this study from the ethics committee of Bielefeld University on the 29 May 2020. This study was conducted in accordance with the guidelines provided by the German Association of Psychology (Deutsche Gesellschaft für Psychologie; DGPs) (Application No. EUB-2020-091). After the participants were provided with information about all pertinent details of the study, its objectives, and the efforts made to ensure data protection and privacy, each participant provided written consent. All the data were processed on a secure network drive located at Bielefeld University in accordance with the General Data Protection Regulation.

### 2.4. Data Collection

We collected data to support this research with the help of an interview guideline. The interview guideline focused on eight topics (see Table 3), and we asked open questions to address these issues. The complete guideline can be accessed at https://doi.org/10.1371/journal.pone.0310137.s002 [17].

The interviews lasted between 1 and 3 h each and were conducted in Italian, which was the mother tongue of the participants. All the interviews were performed online via the Zoom platform by the first author, who was of Italian origin, fluent in Italian and resided in the Italian-speaking part of Switzerland. As a result of restrictions on entry to Italy that were implemented during the COVID-19 pandemic, all the interviews had to be conducted remotely.

These interviews represent part of the PhD project of the first author, who held a bachelor’s degree in occupational therapy and a master’s degree in public health and had experience conducting qualitative research. The second author had a doctorate in the social sciences and extensive experience conducting qualitative research on health services in other countries. She was the supervisor of the first author’s PhD project. The authors had no prior knowledge of the participants in this research.

### 2.5. Data Analysis

The interviews were audio recorded, transcribed verbatim and in their entirety with the assistance of the software program “easytranscript version 3.8.1” (e-283 werkzeug) and anonymised. VERBI GmbH’s MAXQDA 12 software and Word documents were used to facilitate the coding process.

To identify and analyse the facilitators of and barriers to CP and CE promotion, we conducted an inductive qualitative content analysis in line with the suggestions of Kuckartz and Rädiker [22]. Initially, the first author read the material numerous times to obtain an overview of the main aspects of the facilitators and barriers and took notes during this process. She subsequently created an initial coding system that included various themes. The two authors discussed the coding system and applied it to the texts until they reached a consensus concerning the final coding system. This system was then applied to all the interviews by the first author. The first author wrote a detailed interpretation of the interviews that was discussed and revised by the team of authors multiple times until agreement was reached.

### 2.6. Trustworthiness of the Study

We employed distinct techniques to increase the trustworthiness of our qualitative research. To decrease potential bias in the data collection, analysis, and interpretation processes, we used researcher triangulation. The two authors had different and complementary expertise that was helpful for this research (e.g., the first author had knowledge of the Italian health care system, the second author had knowledge of nursing science, and both had expertise in public health and PHC as well as qualitative research methods). Throughout the research process, we employed a reflective approach that involved creating memos and notes that reflected the researchers’ thoughts, assumptions, and preconceptions. Furthermore, by selecting participants with diverse professional backgrounds who could offer comprehensive information about CP and CE in health centres from local, regional, and national perspectives, we accomplished the goal of triangulating resources. In addition, as the interviews were conducted remotely, flexible and safe access to the interviews was ensured. This was particularly important for the interview partners who worked in PHC during the COVID-19 pandemic. This allowed us to collect rich and detailed data (1 to 3 h interviews) despite the difficult circumstances. The first author’s experience in conducting interviews and good preparation helped to prevent technical problems and enabled the interviews to be conducted as planned. Memo writing during the online interviews helped the interviews continue when minor interruptions occurred (e.g., the interview partner was interrupted by a colleague, or the internet connection was unstable for a few seconds).

## 3. Results

The interview partners reported several facilitators and barriers associated with CP and CE in the context of the health centres. We identified four themes that ranged from a focus on contextual, cultural and structural issues to an emphasis on more specific operational aspects. Table 4 presents an overview of the final themes and codes (without frequency claims).

### 3.1. The Culture and Mindset of Practitioners and Organisations as Well as the Competencies That Enable Them to Support Community Participation and Empowerment

The interview partners emphasised that policies and guidelines that prescribe ways of supporting health promotion as well as CP and CE are already in place in the health care system. However, “professional cultures and the actions of individuals are not at that level and do not get there by law” (Practitioner-2_par.17). The lack of a culture and mindset rooted in community-oriented approaches among practitioners and their organisations was identified as a substantial barrier to efforts to promote CP and CE. Therefore, many interviewees called for a “change in culture and organisational structure” (Stakeholder-9_par.70). These two types of change must be linked to one another and synchronised to achieve genuine change.

In this context, practitioners and managers associated with local health care organisations, such as CHCs and LHCTs, served as central actors with regard to this change by learning and establishing a new working culture in the field of health care alongside the community. An important step in the process of changing culture involves raising awareness that health centres are public spaces and, as such, that they are responsible for promoting “collective health” (Stakeholder-17_par.61). This goal requires a perspective that views health as a collective good that is created through joint efforts with the community; in this context, needs are addressed and solved on a joint basis. All the actors involved in this process must have a vision of the co-construction of health and must believe in the value of participatory and empowering approaches before they can work with communities. Some interviewees proposed a change in mindset from “inside” LHCTs and CHCs.

“The Casa della Salute is a hotbed of ideas, so it is the ideas that are born within the Case della Salute that promote its spirit. Otherwise, it’s a house with professionals inside who are doing the exact same thing that they were doing [before], but that’s not the spirit, the mission of the Case della Salute. And this, if one understands it, greatly develops the possibility of empowerment, of developing—of promoting health, of health promotion”.

(Practitioner-11_par.81)

This aspect is, however, limited by the presence of a professional and citizen culture that is rooted in delegation. As one interview partner noted, health practitioners and health care organisations perceive themselves as the “absolute protagonist of the health project for every person” (Stakeholder-7_par.30) and feel that they are the only individuals who are responsible for curing and caring for the people. However, communities and society as a whole have also learned to delegate their health and their responsibility for their health to the health care system.

“[...] for the past 50 years, we have constructed an idea of welfare that is an idea of delegation. And delegation is necessarily devoid of any participatory logic. ‘I mean, I told you to do it, then what do you want from me? You do it’. This is, in my opinion, a very relevant cultural fact that needs to be questioned”.

(Stakeholder-4_par.91)

To raise awareness of the potential of CP and CE and to redefine the mindsets of practitioners and organisations (as well as the community), some interviewees viewed a paradigm shift as essential. The health care system as a whole, as well as health care organisations and consequently practitioners, must shift from a biomedical approach to a paradigm that is both oriented towards a biopsychosocial approach and more people- and relationship-centred. This goal, however, virtually requires a “Copernican Revolution of the health care world” (Practitioner-14_par.144).

“Working on the Case della Salute, if the Case della Salute are characterised by a health paradigm—by a different health paradigm—they also need professional epistemological models that are more adaptive to the new model. Therefore, the effort is not to add the new; the effort is to redefine the old, what each of us carries within. Because the levels of resistance start from there, so the training in the Case della Salute must take this into account and, before inserting new information that, by golly, everyone agrees. I have never found anyone—a politician, a professional— saying that the idea of the Case della Salute is wrong. On a cultural level, there is full support; it is what comes afterwards that is critical. Because the mental models that have characterised us in our history are the ones that we bring into the new context. And if there is no attempt to restructure them and to find better ways of adapting to what is a new frame of work, you will do nothing more than replicate the way you did things before: precisely polyclinic structures where everyone has their own office; maybe you have coffee together, but nothing more”.

(Stakeholder-7_par.101)

According to some interviewees, the biomedical paradigm, which currently strongly influences the mindset of practitioners and health care organisations, is characterised by a historically rooted focus on hierarchies and an authoritarian approach towards patients: “[…] it is you who does not know. I know that you are sick; I have the science, and I cure you” (Practitioner-1_par.50). This approach reinforces power differences between practitioners and the community and is therefore a major limitation to CP and CE processes. Additionally, this mindset is characterised by a fragmented view of health: “I am a nurse; I do this, and it ends there. I did my little piece” (Practitioner-18_par.95). The practitioners are not familiar with a global perspective on health that focuses on, for example, social determinants of health and involves people in decision-making about their own health, and they have not received training in this approach.

This professional culture and mindset are also evident in the strong focus and corresponding pressure on the delivery of health services exhibited by decision makers and managers associated with health care organisations such as CHCs and LHCTs as well as practitioners themselves. With respect to CP and CE, this “service performance culture” (Stakeholder-7_par.30) represents a significant obstacle.

“[...] the thinking behind the Case della Salute, that is, the idea that within a health facility that mainly provides services... that is, there is no longer the time, you understand, there is no time, there is so much hunger to provide these services, to provide visits, to provide services, etc., that it is very difficult to stop for a moment and say, okay, let’s meet, let’s talk...”.

(Practitioner-10_par.30)

The approach taken by certain CHCs towards community partners, such as voluntary associations, demonstrates how problematic this orientation can be. These community partners are often viewed as providers of low-cost services rather than genuine partners.

“[...] the perception that health professionals have [...] that if I say to a nurse coordinator of the Case della Salute, ‘There are volunteers to do this thing,’ her translation is, ‘From tomorrow, I, every day, at all times, I have a volunteer who does this thing.’ But it doesn’t work like that”.

(Practitioner-2_par.62-63)

To promote a cultural shift towards a more people-centred and integrated vision of health, the interview partners viewed the training of all involved actors, especially those from the health care sector, as crucial. Most of the interview partners agreed with the claim that decision makers, managers and practitioners often lack training and competencies pertaining to the value of CP and CE, ways of facilitating these processes, and the appropriate manner in which to approach and collaborate with the community and its representatives, understood as the third sector. A critique raised by some interview partners pertained to the fact that university curricula and continuing training in different health and social professions do not dedicate time to community work. This is, however, important as there is a risk of using the terms CP and CE as “passe-partout word[s]” (Stakeholder-17_par.55)—that is, as approaches that are now in fashion and should be applied but whose “profound sense” and implications are not grasped (Stakeholder-4_par.23). Therefore, the interview partners viewed the change in university curricula as well as the provision of continuing training in CP and CE as primary facilitators of efforts to change “professional paradigms” (Stakeholder-4_par.89). Some interview partners reported positive experiences with the task of imparting the meaning of CP and CE on the basis of “an education action, an experiential training” (Practitioner-1_par.64) in which people can learn and translate what they have learned directly into action.

“[...] a training, not in the sense of the one from the academy, but training in context and within the health system... but these two terms are important. It is one thing to do training within the service network and not within the hospital. […] You have to be inside the service network, and that’s one point. But the other thing is training within the community, with community actors. And you can see the difference: putting multi-professional training inside the service and inside the specific context”.

(Practitioner-15_par.9)

These “trainings [that] are done in the field” (Practitioner-11_par.89) represent major facilitators of CP and CE as they allow practitioners to develop practical competencies directly, such as by offering spaces for communication both in and among groups, building networks, fostering decision-making capacity and revealing individual solutions to a variety of local needs alongside the community.

Interview partners noted that rather than becoming experts in participatory techniques, it is important to acquire a new mindset that enables one to develop professional and organisational routines on the basis of collaboration, relationships and co-decision-making with communities. A main facilitator in this context is the support of practitioners, who may include professional facilitators or experts in CP and CE. According to the experience of some interview partners, especially when CHCs begin working with the community for the first time, these professional facilitators can provide tremendous help in terms of concepts and methods. Regional projects, such as the Community Lab or the CasaLab, have proven to be very fruitful in this context. In these projects, the actors are involved in the implementation and management of projects with the local community.

“[...] from [resolution] 2128, for the realisation of the real objectives of the Casa della Salute, we were supported by a group of trainers who gave us a bit of the methodology, who identified what themes could be addressed. Then, according to the various Case della Salute, we identified a theme, and we went on like this, planning together with them, helped by them, until our project was implemented. [...] three groups were created because Emilia-Romagna is quite large, and therefore the north, centre and south, [and] let’s say the eastern area. And for each one, we identified Case della Salute that had various projects, so to try to spread this methodology and this approach as much as possible. And I must say that this has really led us to a cultural change in our approach to daily life”.

(Practitioner-6_par.57)

Moreover, this learning can be supported by a motivational atmosphere within LHCTs or CHCs because, as one interviewee remarked, practitioners play a critical role in changing cultural processes within the organisation.

“[…] so, there can be a motivating climate for the operators who are in the Case della Salute because they have to do things... that is, they are the ingredients of cultural change, right? Therefore, a strong direction on the part of the management, facilitating conditions that reduce the anxiety experienced by the operators with respect to the change with coherent meaningful policies... The fact of having to subdivide the changes into measurable objectives that are delimited in time, specific, such as projects... like that of [small town1], of CasaLab... that is, a mix of things that are also present in the theory of cultural changes in the organisations”.

(Practitioner-14_par.164)

### 3.2. Reframing Local Health Care Politics, the Organisation and the Role Played by Health Care Organisations in This Context

The interview partners emphasised that the political context in which CP and CE processes occur is crucial as “the relationship between health and politics is a very strong one” (Practitioner-15_par.22). In the context of local health care politics and administration, the values and visions of the ruling parties—and occasionally even of single people in decision-making positions—influence the process of collaboration with the community and third sector and the extent to which CP and CE are promoted.

“[…] there are some municipal administrations that have the ability to strongly involve the associations and others that are more detached from the associations or are in conflict with most of the associations because they do not belong to the political party with which most of the associations identify. So it is clear that where there are municipal administrations that have a... good relationship with the associations, it is possible to have workshops that are really very participatory and others in which involvement is perhaps more limited to those associations that feel that they have a direct interest in the management of the health system”.

(Practitioner-14_par.36)

In general, some interview partners noted that the organisation and top-down management of local health care organisations, such as LHCTs and health centres, represent a primary obstacle and conflict with CP and CE, which are characterised by a more bottom-up approach.

“Because community participation, if it is viewed as a process of empowerment and emancipation of the community that increases its agency and thus also its capacity to choose paths, has entered into conflict with the top-down, politicised organisation [...]”.

(Practitioner-15_par.13)

Furthermore, certain interview partners drew attention to the fact that LHCTs are accountable to health centres in terms of their adherence to managerial principles pertaining to the financial objectives, efficacy, and cost-effectiveness of health care services. LHCTs and CHCs are expected to adhere to these principles, which represent the current technology, bureaucracy, and economy-driven nature of the health care system. As noted critically by some interviewees, the ways in which LHCTs and CHCs are organised contradict approaches that emphasise CP, CE, and well-being. The focus of such organisations is often the number of services they provide instead of indicators that can be used to measure people’s health and well-being.

“If we keep asking our professionals, ‘How many services have you done with respect to... I don’t know, electrocardiography, right? In the budget, I tell you to do 1000, and you, at the end of the year, you did 1000; the next year, I assign you 1100.’ But that is not the question. The question should be —and I tell you this because what it says in the law is community welfare— ‘But how is your community?’”.

(Stakeholder-4_par.28)

The interview partners highlighted the need to change health indicators and outcomes and to facilitate CP and CE to achieve better health outcomes. Therefore, the “normative setup” (Practitioner-15_par.48) of LHCTs and health centres requires a change in the way services are managed and evaluated to meet the genuine needs of the community. However, as long as LHCTs and CHCs must follow the managerial principles mentioned above and have little room to manoeuvre, the interview partners viewed this situation as a serious barrier to efforts to promote CP and CE processes. In this context, the “clumsiness” (Practitioner-3_par.82) of large organisations such as LHCTs with regard to changes to working processes also represents an important obstacle. Another aspect that highlights the difficulty of supporting CP and CE in the context of a bureaucratised system is the provision of freely usable budgets for communities. It can be challenging to provide budgets that citizens can use for projects that they develop on the basis of CP and CE processes when policies and procedures do not account for this type of funding.

“Then it’s clear that, especially in a public context like the Italian one, the regulations are such that there are always procedural constraints for spending the money, which are quite an obstacle to this type of [community participation and empowerment] process, because then the citizens also decide how to spend public money. And not having been elected, that is, not having a specific charge, that’s a problem. But in short, within the projects, you manage to find some small funds that are then contracted and are put in the hands of the citizens”.

(Practitioner-14_par.12)

A main facilitator of efforts to promote CP and CE, as described by the interviewees, is intersectoral collaboration with different local actors who are committed to a common cause.

“[…] all the institutions that are involved in the production of health, namely, certainly health care but also social services, the local authority, the school, the various cooperative associations, etc., must come together to develop a strategy”.

(Practitioner-10_par.53)

This approach is particularly helpful when these intersectoral and interprofessional collaborations predate specific projects and are rooted in a “shared narrative” (Stakeholder-7_par.103–104) pertaining to, for instance, the importance and value of CP and CE. Certain interview partners emphasised that the expansion of these collaborations with and within CHCs is even more important and represents a necessary precondition for efforts to facilitate CP and CE. Furthermore, some interview partners noted that it is critical to identify a specific local organisation that can serve as the community’s “reference point” (Practitioner-1_par.46), a place that members visit not only when they are ill but also when they experience health-related needs. Many interview partners claimed that CHCs could play this role, whereas others maintained that other local organisations operating in the health or social sector could do so. The relevant point is that such an organisation within the community can ensure long-term commitment to CP and CE. Moreover, some interview partners argued that a steering board within CHCs that includes community members and/or their representatives may help formalise CP and CE in the long term. As mandated in 2016 by the regional Emilia-Romagna guidelines concerning health centres, CHC boards can represent this specific participatory governance instrument.

“[...] I believe that if, in the board of the Case della Salute, in the board of the management of the Case della Salute—as it was also envisaged by the same organisational resolution [the regional guidelines for the coordination and development of community of professionals and a medicine initiative in the Case della Salute]—there would actually have been implemented the participation of at least some representatives of the citizens of that area, I believe that at least putting the needs of the citizens on the scales, this would be possible, here. So I believe that at least the board device could be important. It is also true that it could only be, let’s say, a façade, a purely formal device. But I, however, believe in democracy a little bit, so it fits”.

(Practitioner-10_par. 19)

Nonetheless, many interview partners agreed that a sustainable expansion of CP and CE approaches in CHCs requires clear political and institutional will, i.e., “a strong political mandate” (Practitioner-3_par.86) on the part of both regional and local authorities (e.g., the regional health care system, municipality, and local administration) as well as local health care organisations. Moreover, CHCs and LHCTs, in addition to local authorities such as the municipality, must be willing to collaborate on community-specific projects. Thus, a clear position regarding the value of CP and CE, its utility, and an explicit message to practitioners are needed; that is, they must “really want to go in that direction” (Practitioner-14_par.167). Interview partners who emphasised these aspects claimed that practitioners tend to change their working methods only if they receive a clear mission statement from their organisations. However, one interview partner noted that this change in organisations “cannot be based only on the sharing of principles, norms, and values—hence, on a culture—but must find its operational translation” (Stakeholder-9_par.58). To ensure that this political choice is operational in practice, it must become “a binding institutional mandate for the entire organisation of the Case della Salute” (Stakeholder-9_par.58). As this interviewee imagined this situation, this entails a region-wide and specific allocation of financial and human resources. CP and CE projects must “[…] appear in the planning tools of health and social welfare activities” (Stakeholder-9_par.58). All relevant organisations, such as hospitals, LHCTs, districts and CHCs, must include in their action plans and budgetary objectives resources that are allocated to these kinds of activities without neglecting the service delivery objectives that these organisations must pursue and the patients whom they must treat. Similarly, other interview partners claimed that to prevent CP and CE approaches from remaining individual projects that receive support only from practitioners and contexts that are favourable to this topic, specific operational guidelines must be implemented to help to spread this approach “in a comprehensive manner” (Practitioner-3_par.9).

### 3.3. Investing Human and Financial Resources and Creating Appropriate Spaces for Community Participation and Empowerment

According to the interview partners, competent and motivated practitioners who have dedicated time to the task of accompanying these processes represent a major facilitator of efforts to promote CP and CE. As a result of high workloads, working routines and time constraints, opportunities to foster CP and CE are often overlooked. Practitioners “are all stripped to the bone” (Practitioner-10_par.31) as a result of the many services that they must provide; accordingly, “there is no room for anything other than that” (Practitioner-10_par.31). Decision makers, managers and practitioners must therefore be aware that promoting CP and CE processes is an important time investment despite the scarcity of economic and human resources. As one participant noted, they must “accept that a significant slice of my work hours and those of my colleague […] [are] dedicated to this project” (Practitioner-3_par.54). Additionally, since CP and CE are embedded in networks that include the community and various other actors, financial and human resources that extend beyond the scope of single projects are needed in this context.

Managers’ and practitioners’ acceptance of the need to invest in CP and CE processes, however, may be challenged by their “[…] indeterminate, uncertain [character]; they have unexpected outcomes and problems; it is hard to quantify them. It is also difficult to quantify how many hours of work the operators have to invest” (Practitioner- 14_par.150). One reason why the time required by these processes often cannot be calculated is that each CP and CE process is specific to the context, resources, and goals that characterise the local situation. Many decision makers therefore continue to make “a lot of investment in increasing the services because they have an immediate result” (Practitioner-6_par.38). Nevertheless, as some interviewees remarked critically, the outcomes achieved in this context often have only a short-term impact. CP and CE processes stand in contrast to this situation as they have the potential to produce wider social and health goals and outcomes; however, these are often visible only in the long term. This situation can, according to some of the interview partners, be concerning to managers and decision makers in the process of calculating their investments as the results of this process might become visible only after several years. Accordingly, all the actors who are involved in this process must preserve a high level of motivation and not be “disappointed if [they] don’t see results right away” (Practitioner-12_par.22). Aspects that can sustain successful CP and CE processes include constancy, patience, time and “a strong understanding that it takes time for something to change” (Practitioner-12_par.22). Another important facilitator of sustained CP and CE processes was the provision of time for practitioners to discuss and reflect on their approaches and to use a joint method to solve problems due to the “unexpected dynamics of the participative process” (Practitioner-14_par.15).

One criticism raised by many interview partners pertained to the fact that CP and CE activities are often designed only as isolated, discontinuous “spots” (Practitioner-18_par.77) or that they aim only to enhance a specific aspect without addressing the need to alter the culture of all the actors involved in this process. Accordingly, they are frequently unsuccessful in the long term.

A major concern expressed by many interview partners focused on ways of rendering CP and CE processes sustainable. They recognised that practitioners and managers must invest resources to maintain constant contact with the community, care for the corresponding networks, and perform “maintenance” (Practitioner-16_par.17). Furthermore, they emphasised the importance of establishing additional spaces for capacity-building and bottom-up approaches in the context of efforts to develop enduring, collaborative partnerships.

Setting aside specific times for CP and CE processes is a key facilitator of efforts to promote these approaches. Some interview partners contended that these processes should be integrated into the daily work schedule. This task would involve constant interaction and exchange with community members and would represent a core aspect of practitioners’ work. This may mitigate the risk that practitioners may view CP and CE processes as optional side work, i.e., something they can do when they have the opportunity (“If I have time, I will do it. If I have time” (Practitioner-1_par.62)). Promoting this approach internally, however, can be challenging when these processes must be implemented in parallel to actors’ “usual” working tasks.

“[...] I mean, these are not trivial commitments, huh? They are not commitments that you say, ‘I’ll do this little project and bring it home.’ […] So there’s the whole world—I mean, everything is going on regardless... this is just something, let’s say, so complementary—that completes, let’s say, the Case della Salute aspect, but there’s also all the rest of the world, in short, that goes on [...]”.

(Practitioner 12_par.31)

Many interviewees argued that the establishment of spaces in which the community and all involved actors can meet and bond with one another represents a fundamental facilitator of CP and CE. When the interview partners emphasised this aspect, they conceptualised spaces as physical settings within CHCs or other places within the community as well as spaces as dedicated moments. They agreed that these settings should be welcoming, “broad, very accessible, where people can and do stay, in a dialogical logic, among themselves but also recognise themselves at ‘home’” (Stakeholder-4_par.17). Some imagined a room containing comfortable furniture and photographs of the community where people can become familiar with each other, organise meetings and spend time without being “driven by a health care motivation” (Practitioner-16_par.74). In this context, it is important to offer possibilities for meetings that extend beyond the normal working hours of the health centre because different subgroups within the community have different living situations and are available at different times (e.g., the working population might be free only in the evening or weekends, pensioners might be free during the day, and children might be free in the afternoon).

### 3.4. Operational Aspects That Facilitate Community Participation and Empowerment

Many interview partners emphasised that a crucial facilitator of CP and CE processes lies in involving people in issues that genuinely matter to them. Despite the apparent simplicity of this recommendation, as the interview partners reported, the community and the third sector are frequently called to participate after decisions have already been made or when organisations or services merely require approval or “consent” (Stakeholder-4_par.42) from the community or the third sector. Therefore, many interview partners highlighted the importance of involving the community in activities that genuinely met their needs rather than those of the organisations, i.e., needs “that are actually recognised by the people who live there” (Stakeholder-17_par.93). As one interviewee stated on the basis of her experience, when community members were asked to participate in activities that were relevant to them, “they came; they didn’t beg, they didn’t run away” (Practitioner-19_par.58). This claim was confirmed by another interviewee who stressed the long-term investment of some community members when they perceived that they could obtain concrete benefits by participating in such processes.

“[B]ut in reality, we have seen that citizens, if they are called just about very concrete things that they understand that have an impact on their well-being, they are there! They even come willingly. And they participate even if you ask them for such a long commitment”.

(Practitioner-3_par.65)

Moreover, as some interview partners noted, the community should be considered “from the beginning to the end” (Practitioner-18_par.83) of each process. CP and CE can be achieved “when people understand that they can make an impact” (Stakeholder-5_par.7) and have genuine decision-making power. As one interviewee noted, people are motivated to participate and invest resources such as their time in these processes when they are assigned a “decisive role” (Stakeholder-5_par.24). When the community perceives that its participation can genuinely lead to change and when it is involved in activities “where it feels useful” (Practitioner-18_par.80), it gains agency and is willing to participate.

“That is, if they think that there is a problem, they point it out; they understand that they are the ones who can solve it. They are given the initial tools to be able to actually implement these things here, and throughout this process, they cultivate relations among themselves, their direct relations with the local authority, their relations with... this thing builds the substratum for... to ensure that afterwards, there are the outcomes, as we said before”.

(Practitioner-14_par.27)

However, according to some interviewees, the community’s level of participation depends on the goals and the context at hand. For example, the decision-making power of the community may differ when the goal of this process is to support the “collective coproduction” (Stakeholder-8_par.17) of services, to “codesign” (Stakeholder-8_par.17) services or projects, or to “allocate resources” (Stakeholder-8_par.17) to specific contexts or services. In any case, as emphasised by the interview partners, the level of CP must be clarified from the outset, as must the role played by each actor within that process.

“[We] try to be clear from the beginning to share with the project actors the level of participation that can be granted in that project depending on the context. So there are certain situations in which it is possible to grant higher levels of control to the community and others in which it is necessary, however, to explain that the role of the project is to bring in the community’s demands, to involve them as much as possible—but then you have to mediate with institutional cases in which the decision-maker […] is different from the person who implements the project. So we try to be clear in terms of our definition of the limits of participation to ensure that those who engage in the project are aware of it and clear about it […]”.

(Practitioner-14_par.7)

Similarly, various interview partners agreed that clear communication on the part of the individuals who initiate this process is an important facilitator of efforts to promote CP and CE processes, which is conducive to the establishment of trust and is fundamental to the task of establishing a relationship with the community. The presence of an individual who is responsible for coordinating this interaction is particularly important. When practitioners and other actors who are involved in this process begin to interact and initiate a dialogue with the community, they must use appropriate language from the outset and must be explicit regarding the goals of the activities that they propose as well as their intentions in the context of promoting a given CP or CE process.

In general, the interview partners noted that it is fundamental to know the community, the diverse subgroups that it includes, their habits, and the local actors, including municipalities, schools, and voluntary associations. Community members who serve as “sentinels” (Stakeholder-17_par.71) or “community connectors” (Stakeholder-4_par.55) and interact with CHCs can be extremely valuable informants and facilitators. Often, these community members know the community well because they work or live in it; thus, they can reveal “subthreshold situations” (Practitioner-6_par.44) that the CHCs and other organisations may not be able to identify. Moreover, practitioners should spend time within the community in an informal way to improve their familiarity with the community:

“They went to bars, they went to markets, and they were there. They were there, being within the community. And we started establishing relationships. […] We would hang out there and build relationships with people”.

(Practitioner-15_par.38)

The interview partners identified solid knowledge of the community as an important prerequisite for CP and CE processes. However, one interviewee mentioned that it can occasionally be challenging to understand which members of the community should be involved in such processes, especially in the context of “dealing with particular issues, not to forget anyone” (Practitioner-6_par.63). Some interview partners highlighted the importance of involving all relevant actors and their different perspectives to obtain a broader overview of relevant needs and possible ways to address them.

In the context of efforts to engage third-sector associations as community representatives, strong motivation and will to work together on the part of such associations represent important facilitators of CP and CE processes. This collaboration becomes even more fruitful when the “third sector [is] competent and proactive” (Practitioner-2_par.89). However, a challenge that some interview partners encountered in the context of their efforts to engage associations in the third sector pertained to focused lobbying exclusively for specific subgroups or even competing interests. This process entailed the risk of receiving only certain opinions and identifying certain needs of specific groups represented by the third sector while overlooking the many different views that characterise such a diverse community.

“[...] some voluntary associations represent organ pathologies; therefore, there is the voluntary association that takes care of patients who have ALS [amyotrophic lateral sclerosis]; there is the voluntary association that takes care of patients who have Alzheimer’s. So this, obviously, can be a disruptive condition with respect to a vision of things together because each of these parties obviously brings its own specific field of interest to its level of attention”.

(Stakeholder-7_par.14)

Accordingly, it is very important “to distinguish, to separate” (Stakeholder-8_par.13) the different associations in the third sector on the basis of their roles and to remain aware of this factor when involving these associations in CP and CE processes.

Moreover, the interview partners identified various community subgroups that are particularly difficult to involve in CP and CE processes, such as adolescents or young adults, certain immigrant groups, the working population and isolated older people. The interviewees believed that involving these allegedly difficult-to-reach subgroups is important as they often have different needs, do not “self-involve” (Practitioner-11_par.38) in such activities and are often not accustomed to participatory processes.

“And so there are mingled … a rainbow, really, of needs, of... desires that, if you don’t offer an equally wide space of possibility to bring them here too, you risk only weaving a very small part of what you call community because it depends on your need. That is, if I obviously go to the meeting at five o’clock in the morning to drink tea, I will only meet people who are at home at five o’clock in the morning, who like to drink tea, who like to be with people in a social way. But I won’t find all the other people. I won’t find the child who does his homework. I won’t come because I don’t like tea; therefore, I won’t come to tea. I won’t find a whole range of other people. Therefore, I have to offer so many different things to have a range of people with whom I can then engage slowly”.

(Practitioner-13_par.26)

This process involves creating opportunities for these different needs to emerge and for people from diverse subgroups to participate. This approach, however, must account for the possibility that members of some more disadvantaged groups will not participate as a result of concerns and worries that are much more pressing and relevant to them (such as young adults seeking jobs or working parents who struggle with their salaries) than the task of considering how their environment can be improved.

According to some interview partners, different community groups must often first become aware of the coexistence of their own needs and the diverse needs of other community members. This awareness is relevant to the task of identifying not only group-specific needs but also collective needs, which can “intersect” (Practitioner-13_par.25) with each other. As one interviewee noted,

“[…] the moment you are able to find a common goal, a synergy, it certainly works out very well. When different needs are not the same or, indeed, sometimes they are a little bit in contrast, of course, things crack a bit—but that is okay, that’s it. I mean, it means that nobody…, you don’t get homogenised and nobody gives in. That’s it. But, let’s say, though, that the goal—keeping the goals broad enough, which is the Casa della Salute, [Casa] della comunità; you hardly go against it”.

(Practitioner-13_par.82)

To allow different community members to meet one another, low-threshold opportunities for meeting in CHCs must be created. In the context of easily accessible community activities (e.g., community parties featuring food, community walks, tea drinking meetings, gymnastics, or local playful activities (with schools) to which all family members can be invited), people who exhibit similar or complementary needs or interests have the opportunity to meet one another and establish relationships. This approach has the potential to facilitate the creation of significant networks, which can subsequently facilitate the provision of mutual support. With respect to these activities, one interview partner made the following suggestion:

“[…] it’s not what improves their health, in my opinion, but it gets them out of the house, gets them to meet each other, gets them sunbathing... so it’s important to us. It encourages them to overcome mistrust, to get to know their neighbours; it creates mutual relationships. Then, when they need it, it’s important”.

(Practitioner-10_par.28)

Therefore, varying the strategies and activities that are used to connect with various community members is critical. This approach is especially crucial in the context of efforts to engage with more disadvantaged or difficult-to-reach groups. Another useful strategy that can be employed to reach such groups is “the involvement of informal leaders who manage to draw in groups of citizens” (Practitioner-12_par.134).

## 4. Discussion

The findings of this study provide new evidence regarding various barriers to and facilitators of CP and CE in the context of PHC centres by revealing that to promote CP and CE, a supportive context must be established at different levels. In the context of Italy’s PHC reform [12], which enforces CP and CE in health centres nationwide, and in light of a current report on the implementation of CHCs that shows that CP and CE approaches are not yet promoted in all Italian health centres [27], these results become particularly relevant because they provide concrete practice and policy implications.

According to our study, this transformation requires (a) changes in the mindset and culture of the entire health care system, including the organisational and operational levels; (b) a reorganisation of the local health care system and the corresponding organisations; and (c) appropriate resources, skills and working methods.

### 4.1. Changes in Mindset and Culture to Support Community Participation and Empowerment

Our analysis revealed the importance of cultural and organisational change in the context of efforts to promote CP and CE processes. Major challenges in this context include the health care system and organisational conditions as well as a mindset that features a narrow focus on service delivery. As one interview partner noted, although CHCs must promote CP, as health care facilities they operate with a mindset that focuses on their obligation to provide services and experience the corresponding pressure, which leaves them with little space to focus on CP and CE approaches. Accordingly, the interview partners highlighted the importance of adopting new mental models and a new vision of health that views CP and CE as fundamental components of people-centred health care and that is in line with or integrated into service provision. In this context, the interview partners advocated for a shift to a biopsychosocial paradigm and a culture that places a stronger focus on collaboration between health organisations and communities. As confirmed by other scholars, hierarchies and power imbalances among relevant actors that result from the historically rooted biomedical paradigm that characterises the health care system must be analysed and challenged [20,28] with the goal of developing new ways of reasoning and working in the PHC context. As our results reveal, the interview partners believed that this mindset and culture must be embraced through cultural change. The literature on organisational cultural change in health care settings highlights the importance of considering not only the visible structures and observable behaviour of the people within an organisation but also their values and beliefs. Moreover, basic assumptions that are often preconscious, such as those pertaining to hierarchies and power within a care relationship, play a significant role in culture change [29]. These assumptions shape the attitudes and daily working habits of the members of an organisation [30,31] and are therefore relevant in this context.

According to the interviewees, continuing training is an important way of changing people’s mindset and culture. Similarly, Johnson and colleagues [30] recommended the use of long-term learning processes, such as workshops, discussions and action planning, to foster organisational change. Our results indicate that changes in attitudes and views concerning CP and CE seemed to be particularly successful when they were supported by facilitators who were experts in the fields of CP and CE and when experiential education and learning environments were employed, which allowed the knowledge and skills acquired in this context to be transferred directly into the practice environment (e.g., CP and CE projects in a specific community setting) and subsequently reflected by the teams themselves. Other scholars have reported similar results and highlighted the importance of proposing moments of exchange among the various actors involved in the learning process, thus enabling them to reflect on these experiences and improve their work [20,21,30,32]. Nonetheless, the interviewees in our study went a step further by requesting modifications to the university curricula for health and social care professionals with the goal of promoting professional education, knowledge, skills and values related to community orientation, CP and CE.

Cultural change pertaining to CP and CE should not be limited by the involvement of only stakeholders and practitioners. When efforts are made to change the culture in health care contexts, it must be remembered that these processes are multifaceted, complex and dynamic. Moreover, these processes are influenced by many factors, such as local subcultures and interactions with actors outside the health care organisation. As such, it is not always possible to manage them exclusively from the inside [29,31]. For stakeholders and managers, the task of changing culture includes involving different relevant local actors, such as those associated with social services and the municipality, as well as the community as a whole (including citizens, the third sector, users and their caregivers) in processes of change [19,20,21,33]. This cooperation is important because the partners of the organisation can serve as enablers or even blockers of cultural change. Furthermore, as some interview partners stressed, there is an expectation among the population that health and care can be delegated or externalised to professionals. It is therefore relevant to invest in efforts to raise community awareness on the potential of CP and to build capacity and create spaces for CE with the goal of fostering cultural change. Changes to organisational culture can take years and require sufficient resources and commitment from the various actors involved in this process. Awareness of possible setbacks and celebrations of successful initiatives are important for maintaining a high level of motivation and a strong sense of progress among all involved actors [30].

### 4.2. Reorganisation of the Local Health Care System and the Corresponding Organisations

With respect to structural-organisational aspects, our results reveal relevant obstacles to CP and CE that pertain to the (top-down) organisation of LHCTs and CHCs. The interview partners criticised the fact that LHCTs and CHCs must follow mainly managerial principles that can impede CP and CE processes and leave little space for participatory, bottom-up approaches to health. Therefore, some interview partners advocated for a reorganisation of the health care system and LHCTs with the goal of rendering them more people-centred and participatory. This recommendation is in line with the strategies included in the WHO’s Framework on Integrated People-Centred Health Services [6]: To make health organisations more people-centred, changes at the organisational, health care system and policymaking levels are necessary. One such strategy focuses on “establishing an enabling environment”, which involves, for example, a reorientation of the workforce, leadership and management in support of change [6]. These aspects are in line with our results and emphasise the importance and influence of (local) politics and the roles played by the leaders and stakeholders of organisations in efforts to support practitioners working in a person-centred manner. If a favourable sociopolitical and economic environment is established with the aim of promoting community orientation and the corresponding values and actions, this approach can help foster CP and CE [6,33]. For this purpose, decision makers or stakeholders must advocate (particularly with respect to the organisation’s members) for a new vision and way of working and must emphasise the relevance of modifying existing approaches. Moreover, they should be able to recruit a critical mass of supporters for change by involving all members in the process of transformation [30]. This approach is important because the introduction of major modifications to an organisation can easily elicit resistance to change [30,31]. In this regard, as our results indicate, a strong organisational and political mandate for CP and CE processes is an important facilitator of advancement. Other scholars have confirmed the importance of a clear and visible commitment on the part of local political and health organisations to endorse practitioners [18,19,33,34].

As our results show, commitment to CP and CE must be reinforced. However, in this context, binding institutional responsibilities that range from the specific allocation of resources to organisational guidelines and statements are necessary. These guidelines should be accompanied by incentives that reward individuals who comply with the new ways of working [30]. Although Decree 77/2022 does not mention specific participatory bodies within the CHCs, it paves, though, the way for different forms of community engagement [12]. This is evident in the Italian national guidelines for the promotion of CP and CE in CHCs which concrete the Decree by proposing various participatory governance instruments and approaches to CP and CE [16]. In addition, earlier regional Emilia-Romagna policies have pointed to the importance of institutionalised engagement forms [9]. In this regard, participatory bodies should be created through the sustained implementation of decision-making groups, such as CHC boards (which are requested by regional health policies [9]), or other governance structures that can involve community members in the decision-making processes of health organisations [35]. Such steering structures offer the opportunity to institutionalise CP in PHC settings. Their composition should be planned thoroughly and should account for the heterogeneity of the community, including the representation of disadvantaged subgroups [36]. This aspect also aligns with the WHO’s strategy for people-centred health systems, which calls for “strengthening governance and accountability” through participatory governance processes [6]. However, the implementation of such governance boards requires relevant actors to address issues related to the influence and decision-making power of the community [37].

As noted by our interview partners, preexisting collaborations and networks with local actors and services serve as fundamental facilitators of CP and CE processes [28,33,35]. In this context, interprofessional and intersectoral collaboration are important facilitators of CP and CE [19]. This approach is in line with the WHO’s Framework [6] strategy, which aims to promote people-centredness by “coordinating services within and across sectors”. This strategy identifies collaboration, including intersectoral partnerships and coordination among different health and social providers, as an important way for health services to adopt a more people-centred focus. Interestingly, our results indicate that interprofessionalism might even be a prerequisite for successful CP and CE and should therefore be promoted in parallel with community-oriented approaches.

In this regard, new opportunities for community involvement may also arise as a result of the reorganisation of the health districts, with a mandate to focus more on population-based, comprehensive community care [12]. In their coordination function across settings, the health districts interact with different services and organisations, such as the municipalities, and may play an important role in facilitating interprofessional and intersectoral collaborations that is beneficial for the creation of joint CP and CE processes. However, it requires that the health districts recognise the value of such approaches and are willing to support them, for example, to understand priorities of the population and, accordingly, create more needs-based services.

### 4.3. Resources, Skills and Working Methods

According to the findings of the current study, major challenges to efforts to promote CP and CE processes persist. These challenges are related to the lack of resources in PHC services, time constraints, and the overall difficulty of promoting these processes, which frequently require an extended period of time. These obstacles have also been described in other studies [19,20,21,33,38,39]. This situation demands a context that can provide ongoing human and financial resources as well as dedicated time for community work and exchange among practitioners, thus allowing for the emergence of meaningful CP and CE and facilitating the long-term integration of these processes into practitioners’ daily work [20,21,28,33,34,39,40,41]. In this context, community members’ access to resources and incentives for their participation in such processes is also relevant and has ethical implications. The participation of community members in participatory governance structures (e.g., boards, health committees, advisory councils) can be sustained through material and immaterial incentives such as transport reimbursement, small amounts of cash or gifts, certificates, public recognition, training, meals or social events for the participants [33,42]. Incentives and compensation are important to ensure the sustainability and retention of members because they have opportunity costs (e.g., time) [42]. Moreover, compensation in the form of payments might facilitate the participation of people with low incomes, thus promoting equal representation of different community interests. However, depending on the type of incentives and the amount of pay, this can also threaten neutrality in involvement and motivate participation for the sake of the payment. Therefore, compensation should be fair, appropriate and realistic with respect to the given resources [42].

In this context, it is crucial to understand and assert, by challenging relevant decision makers, that CP and CE processes are highly specific to the local context in terms of resources and goals; therefore, no “one size fits all” approach can be used [43]. The establishment of enduring, collaborative partnerships requires time and investment in the capacity building of all actors involved in this process to ensure that the resulting changes are sustainable.

Our results reveal that a fundamental element of this process is knowledge of the community, its subgroups, and local actors, such as those from the third sector. In line with our findings, other studies have discussed the importance of practitioners’ efforts to spend informal time in the community to obtain a genuine understanding of that community [32] and establish social bonds [41]. Another result of this study suggests the importance of involving people in issues that genuinely matter to them. The interview partners’ experiences revealed that people are willing to participate, even for longer periods, if the topics at hand are relevant to their lives and if they or their community can obtain direct benefits through such engagement.

In this regard, it is important that practitioners and stakeholders who are willing to involve communities understand which issues are important to the communities and can choose from a variety of engagement models. Depending on the objectives, longer-lasting and formalised forms of engagement (e.g., in steering committees/boards of the CHCs) or time-limited forms of CP (e.g., in panel discussions) can offer different ways to reveal unexplored needs, identify community priorities and decide on relevant actions to take [16]. Future research and evaluation should focus on this area to determine which groups are reached through which methods and engagement models. However, it remains fundamental that people’s motivation to participate is high when they observe that they can have a genuine influence and can exert decision-making power over relevant processes and actions. The literature has emphasised the insufficiency of only listening to what people need; rather, people must be involved in different stages of the CP process, including the possibility of exerting control over the actions and consequences associated with that process [33,35,44,45]. This finding is closely related to issues of power and empowerment because developing the community’s capacity as well as its ability to wield power or to share it with other relevant actors allows it to exert a genuine influence on meaningful aspects of their lives [44,45,46]. In this regard, according to the interview partners, a relevant way to sustain the CP and CE processes is through clear communication, for example, regarding the level of participation; the roles, goals and intentions of involved actors; and their coordination. In line with these findings, other studies have focused on the relevance of transparency, efforts to establish relationships and build trust, and information sharing to create significant CP processes [28,33,35].

A major challenge is the task of understanding who should be involved in CP and CE processes. Although the results of our study highlight the importance of involving different community actors in this context, a major concern raised by the interview partners is the role of the third sector as a representative of the community. Our results reveal that the engagement of the third sector is viewed as crucial to successful approaches to CP and CE; however, as indicated also by other scholars, the appropriate representation of the community, which is frequently heterogeneous and consists of different community subgroups, can be problematic [21,28]. In particular, as partially supported by our results, it can be difficult for disadvantaged or specific community groups to be involved in such processes, for example, as a result of challenging life situations [38,45]; the feeling that they do not belong to the community, which decreases their interest in participating in such activities [45]; their potential adherence to certain values or experiences of marginalisation [47]; or their internalised belief that they are powerless regarding the context in which they live [45,48]. It is therefore important to understand the characteristics of these groups and the barriers to their involvement [47]. The interview partners proposed a variety of low-threshold community activities (including the involvement of informal community leaders) that may enable them to engage with these groups more effectively. A favourable context with appropriately timed activities and accessible and familiar spaces featuring an informal atmosphere and adequate communication methods are some of the ways these groups can be reached [35]. Moreover, it is crucial to build capacity and create opportunities for the development of CE as well as to establish relationships between these groups and CHCs to ensure that they are able to participate in CP processes [47].

### 4.4. Limitations of the Study

We draw on the experiences and views of interviewees who have expert knowledge based on practical experience with CP and CE processes coming from a sample of good practices, and experiences with the management of and research on these processes in the context of CHCs in Emilia-Romagna, Italy. It would have been valuable for this research to include the communities’ views on the relevance of and experiences with CP and CE processes to complement the understanding of the facilitators and barriers, particularly for microlevel processes. This issue should be addressed in future studies.

We used purposive and snowball sampling to help identify interview partners from many of the CHCs that, according to experts and the literature on this topic, are good-practice examples of promoting CP and CE. However, we cannot exclude the possibility that there were more health centres that may also have been relevant for this research. Moreover, we used this sampling strategy to reach out to participants who were confronted with and sensitive to the topic. The inclusion of participants with no experience and health centres that do not develop CP and CE would have resulted in different barriers and facilitators. However, considering that the support of CP and CE is now mandatory by law in all CHCs, our results make an important contribution to this topic.

Another limitation concerns our data on CP in the context of physical distancing. This topic was discussed with the interview partners, as data were collected during the COVID-19 pandemic. However, most of the interviewees reported that the projects were temporarily halted during the worst moments of the COVID-19 pandemic. Because we did not have much data on this topic, we decided not to include these data in the results. Moreover, if interviews had been conducted during times without COVID-19, we might have received more comprehensive narratives.

## 5. Conclusions

By introducing CHCs, Italy has followed recommendations to strengthen its PHC system by increasing its people-centeredness and community orientation. Our findings contribute new evidence to the literature on this topic with regard to the facilitators and barriers that stakeholders and practitioners encounter in their efforts to promote CP and CE processes in primary care settings. We identified different levels that must be considered in this context and that are often interconnected: Overarching contextual and structural aspects, such as the culture and mindset of (health) organisations and their members; local politics; the organisation of the health care system and corresponding organisations; resources and spaces; and specific skills and working methods.

Our study provides suggestions that can help stakeholders, managers and practitioners consider the different facilitating and challenging aspects that characterise this situation at different levels. For example, stakeholders and managers should be aware of the aspects that can influence cultural and organisational change (e.g., historically rooted hierarchies and a biomedical paradigm that challenges CP and CE) as well as various ways they can help support this change (e.g., continuing action-learning on the part of all actors involved in this process). They must also be aware that the promotion of CP and CE processes requires a favourable sociopolitical and economic environment and that they must exhibit a clear commitment in this context through the allocation of resources, the incorporation of CP and CE processes into local health plans and the institutionalisation of governance structures that include community members. In this regard, the first Italian national guidelines for CP and CE development in CHCs, which provide examples of governance instruments such as instruments for strategic planning, citizens’ committees, and citizens’ tables [16], can be of great value but must be implemented with consideration of the objectives of each CP and CE process and the (local) cultural and organisational context.

Moreover, human and financial resources must be made available, including sufficient time for community work and exchange among practitioners as well as spaces to establish social bonds and relationships with the community and relevant actors (e.g., those from the third sector). Preparations must be made to involve the community by paying attention, for example, to the need for clear communication, proposing activities that are meaningful to the target population, and considering how to involve representatives of the community (e.g., actors from the third sector) and how to reach out to disadvantaged groups through low-threshold approaches.

## Figures and Tables

**Table 1:** Characteristics of community health centres (CHCs) in Italy.

Population	
Hub CHC	40,000–50,000 inhabitants
Spoke CHC	Fewer than 40,000 inhabitants (depends on the local territory) Various smaller spoke CHCs refer to one larger hub CHC
**Professionals**	
Hub CHC	General practitioners, paediatricians, nurses, social workers, specialists (e.g., cardiologists and diabetologists), and administrative personnel. Some CHCs also include psychologists, midwives, and prevention and rehabilitation professionals (e.g., physiotherapists).
Spoke CHC	General practitioners, paediatricians, nurses, specialists, and administration personnel

Sources: [12,15].

**Table 2:** Characteristics of the sample (N = 19).

Participant Characteristics	Number of Participants
**Participants, total**	**19**
**Practitioners**	**13**
Community Health Centre staff involved in the coordination and/or creation of community participation and/or community empowerment processes in a Community Health Centre	5
Cooperating professionals from a local health care agency or social services who were involved in the coordination and/or creation of community participation and/or community empowerment processes in a Community Health Centre	8
**Stakeholders**	**6**
Representatives of third sectors at the national level	2
Researcher and project coordinatorsof community participation and/or community empowerment processes in Community Health Centres/primary health care at the national or regional level	2
Managers of local primary health care/Community Health Centres	2
**Gender**	
Male	9
Female	10
**Professional background**	
Nurse	3
Physician	8
General practitioner (specialisation in general medicine)	3
Other (e.g., specialisation in hygiene and prevention)	5
Pedagogue	2
Other (e.g., studies in health management, psychology, economics)	6
**Focus of expertise**	
Local level	14
Regional level	2
National level	3

Source: Reproduced from: Luisi, D.R. & Hämel, K., “Understandings of community participation and empowerment in primary health care in Emilia-Romagna, Italy: A qualitative interview study with practitioners and stakeholders”, PLOS ONE, 2024, https://doi.org/10.1371/journal.pone.0310137 [17]; Table DOI: https://doi.org/10.1371/journal.pone.0310137.t001 (accessed on 9 May 2026), licensed under Creative Commons Attribution 4.0 (CC BY 4.0).

**Table 3:** Topics included in the interview guideline.

Topics Included in the Interview Guideline
Development of the Case della Salute in recent years Understanding of CP and CE Estimations of the relevance and consequences of CP and CE Experiences with approaches to CP: Examples and descriptions of processes/activities Challenging and supporting aspects of CP/CE CP in the context of physical distancing Future developments

**Table 4:** Inductive themes and codes for the facilitators and barriers associated with efforts to promote CP and CE.

Themes	Codes Pertaining to the Facilitators and Barriers Associated with Efforts to Promote CP and CE
The culture and mindset of practitioners and organisations as well as the competencies that enable them to support community participation and empowerment	Lack of a culture and mindset with a community orientation among practitioners and organisations Changes in culture/mindset Concept of health as a collective good Practitioners and managers of health care organisations as central players in cultural and organisational change Culture of delegating health to services Paradigm shift Biomedical approach, hierarchies, and power imbalances Fragmented view of health Focus and pressure on service delivery New mental models and a new vision of health Knowledge of and competencies pertaining to CP/CE Training and education
Reframing local health care politics, the organisation and the role played by health care organisations in this context	(Local) politics influence the possibilities of CP/CE Organisation of the health care system: Agencies’ focus on cost-efficiency, management principles Bureaucratisation of processes Intersectoral and interprofessional collaborations CHC boards Clear political and institutional will and commitment Reorganisation of the health care system and the LHCTs
Investing human and financial resources and creating appropriate spaces for community participation and empowerment	Dedicated, motivated, and competent practitioners High workloads, working routines and time-constraints Financial, human and time resources Difficulty of changing working processes in large organisations Sustainability of CP and CE processes Spaces for CP/CE
Operational aspects that facilitate community participation and empowerment	Involvement of people in issues that genuinely matter to them Real decision-making power and a decisive role Level of participation Clear communication Responsible coordinator Knowledge of the community Engagement of third sector associations Efforts to access hard-to-reach subgroups Creation of opportunities for different needs to emerge and people from diverse subgroups to participate; discovery of collective needs Mediators/informal community leaders/intermediaries from the community

## Data Availability

The data presented in this study are available on request from the corresponding author due to privacy and ethical restrictions.

## Abbreviations

The following abbreviations are used in this manuscript:

PHC	Primary health care
CP	Community participation
CE	Community empowerment
CHC	Community health centre
LHCTs	Local health care trusts

## References

[1] OECD (2020). Realising the Potential of Primary Health Care.

[2] OECD, European Union (2016). Health at a Glance: Europe 2016: State of Health in the EU Cycle.

[3] World Health Organization Regional Office for Europe (2018). From Alma-Ata to Astana: Primary Health Care—Reflecting on the Past, Transforming for the Future. Interim Report from the WHO European Region.

[4] Rouleau K., Rajan D., Winkelmann J., Kringos D., Jakab M., Khalid F., Lessof S., Porignon D., Schmets G., Rajan D., Rouleau K., Winkelmann J., Kringos D., Jakab M., Khalid F. (2024). The PHC approach: An introduction. Implementing the Primary Health Care Approach: A Primer.

[5] Starfield B., Shi L., Macinko J. (2005). Contribution of Primary Care to Health Systems and Health. Milbank Q..

[6] World Health Organisation (2018). Continuity and Coordination of Care: A Practice Brief to Support Implementation of the WHO Framework on Integrated People-Centred Health Services.

[7] Rifkin S.B. (2014). Examining the Links between Community Participation and Health Outcomes: A Review of the Literature. Health Policy Plan..

[8] Wallerstein N. (2006). What Is the Evidence on Effectiveness of Empowerment to Improve Health? Health Evidence Network Report.

[9] Luisi D., Hämel K. (2021). Community Participation and Empowerment in Primary Health Care in Emilia-Romagna: A Document Analysis Study. Health Policy.

[10] Ministero della Salute (2007). Decreto Ministeriale 10 July 2007. Progetti Attuativi Del Piano Sanitario Nazionale—Linee Guida per l’Accesso al Cofinanziamento Alle Regioni e Alle Province Autonome Di Trento e Bolzano. Legge n. 296/2006.

[11] Brambilla A., Maciocco G. (2022). Dalle Case Della Salute alle Case di Comunità.

[12] Ministero della Salute (2022). Decreto Ministeriale 23 May 2022, n.77. Regolamento Recante La Definizione Di Modelli e Standard per Lo Sviluppo Dell’assistenza Territoriale Nel Servizio Sanitario Nazionale. (22G00085).

[13] De Maeseneer J., Poplas Susic A., Wolfe S.A., Qingyue M., Moosa S., Raskin L., Symondson T., Hawkins D.R. (2019). Community Health Centres: Operationalizing the Declaration of Astana on Primary Health Care.

[14] Poplas Susic A., Aaerendonk D., De Maeseneer J., Dedeu T. (2026). Community Health Centres as a model of care: Contextualizing for Europe—A Delphi study. Prim. Health Care Res..

[15] Agenzia Nazionale per i Servizi Sanitari Regionali (2024). Linee di Indirizzo per l’Attuazione del Modello Organizzativo delle Case della Comunità Hub. https://www.agenas.gov.it/images/agenas/PNRR/Linee_di_indirizzo_CdC.pdf.

[16] Agenzia Nazionale per i Servizi Sanitari Regionali (2023). Documento di Indirizzo Contenente Indicazioni per la Promozione Della Partecipazione/Co-Produzione dei Pazienti, dei Cittadini e Della Comunità nell’Ambito Delle Case Della Comunità. https://data.agenas.it/web/documenti/01_Documento_di_Indirizzo_partecipazione_co-produzione_CdC_Dic.2023.pdf.

[17] Luisi D.R., Hämel K. (2024). Understandings of Community Participation and Empowerment in Primary Health Care in Emilia-Romagna, Italy: A Qualitative Interview Study with Practitioners and Stakeholders. PLoS ONE.

[18] Gervits M., Anderson M. (2014). Community-Oriented Primary Care (COPC) in Barcelona, Spain: An Urban Copc Experience. Int. J. Health Serv..

[19] López Torrent E., Forcada Vega C., Miller F., Pasarin Rua M.I., Foz Gil G. (2010). Factores que facilitan y dificultan el desarrollo de los proyectos comunitarios. Estudio observacional de la red AUPA de centros de atención primaria de Cataluña. Aten. Primaria.

[20] MacFarlane A.E. (2020). Optimising Individual and Community Involvement in Health Decision-Making in General Practice Consultations and Primary Care Settings: A Way Forward. Eur. J. Gen. Pract..

[21] McEvoy R., Tierney E., MacFarlane A. (2019). ‘Participation Is Integral’: Understanding the Levers and Barriers to the Implementation of Community Participation in Primary Healthcare: A Qualitative Study Using Normalisation Process Theory. BMC Health Serv. Res..

[22] Kuckartz U., Rädiker S. (2023). Qualitative Content Analysis: Methods, Practice and Software.

[23] Flick U. (2009). An Introduction to Qualitative Research.

[24] Meuser M., Nagel U., Bogner A., Littig B., Menz W. (2002). ExpertInneninterviews—Vielfach Erprobt, Wenig Bedacht. Ein Beitrag Zur Qualitativen Methodendiskussion. Das Experteninterview. Theorie, Methode, Anwendung.

[25] Luisi D.R., Hämel K. (2026). How are Community Participation and Empowerment Processes Shaped and Fostered in Practice in Community Health Centres in Emilia-Romagna, Italy? A Qualitative Study. Prim. Health Care Res. Dev..

[26] Patton M.Q. (1990). Qualitative Evaluation and Research Methods.

[27] Agenzia Nazionale per i Servizi Sanitari Regionali (2026). Report Nazionale di Sintesi dei Risultati del Monitoraggio dm 77/2022 II Semestre 2025. https://www.agenas.gov.it/images/Report_Nazionale_DM77_II_semestre_2025_-_080426.pdf.

[28] Crowley P. (2005). Community Participation and Primary Care: Learning from the Building Healthy Communities Programme; Programme (Combat Poverty Agency).

[29] Mannion R., Davies H. (2018). Understanding Organisational Culture for Healthcare Quality Improvement. BMJ.

[30] Johnson A., Nguyen H., Groth M., Wang K., Ng J.L. (2016). Time to Change: A Review of Organisational Culture Change in Health Care Organisations. J. Organ. Eff. People Perform..

[31] Mannion R. (2022). Making Culture Change Happen.

[32] Wang M., Perzynski A., Ronis S. (2023). Bringing Community Oriented Primary Care into an Academic Training Setting: A Qualitative Study. Prev. Med. Rep..

[33] Erku D., Khatri R., Endalamaw A., Wolka E., Nigatu F., Zewdie A., Assefa Y. (2023). Community Engagement Initiatives in Primary Health Care to Achieve Universal Health Coverage: A Realist Synthesis of Scoping Review. PLoS ONE.

[34] Abelson J., Forest P.-G., Eyles J., Casebeer A., Martin E., Mackean G. (2007). Examining the Role of Context in the Implementation of a Deliberative Public Participation Experiment: Results from a Canadian Comparative Study. Soc. Sci. Med..

[35] National Institute for Health and Care Excellence (2016). Community Engagement: Improving Health and Wellbeing and Reducing Health Inequalities. NICE Guideline.

[36] Leyns C., Willems S., Powell R.A., Camacho V., Fabrega R., De Maeseneer J., Rawaf S., Mangtani P., El-Osta A. (2023). From Disease- to People-Centred Pandemic Management: Organized Communities, Community-Oriented Primary Care and Health Information Systems. Int. J. Equity Health.

[37] Law K.L., Saunders J.A. (2016). Engaging Consumer Voices in Health Care Policy: Lessons for Social Work Practice. Health Soc. Work.

[38] Gholipour K., Shokri A., Yarahmadi A.A., Tabrizi J.S., Iezadi S., Naghibi D., Bidarpoor F. (2023). Barriers to Community Participation in Primary Health Care of District Health: A Qualitative Study. BMC Prim. Care.

[39] Tierney E., McEvoy R., Hannigan A., MacFarlane A.E. (2018). Implementing Community Participation via Interdisciplinary Teams in Primary Care: An Irish Case Study in Practice. Health Expect..

[40] Cleary S., Schaay N., Botes E., Figlan N., Lehmann U., Gilson L., Padarath A., King J., English R. (2015). Re-Imagining Community Participation at the District Level: Lessons from the DIALHS Collaboration. South African Health Review 2014/15.

[41] Collins P.A., Resendes S.J., Dunn J.R. (2014). The Untold Story: Examining Ontario’s Community Health Centres’ Initiatives to Address Upstream Determinants of Health. Healthc. Policy.

[42] Chumo I., Kabaria C., Oduor C., Amondi C., Njeri A., Mberu B. (2023). Community advisory committee as a facilitator of health and wellbeing: A qualitative study in informal settlements in Nairobi, Kenya. Front. Public Health.

[43] Montesanti S.R., Abelson J., Lavis J.N., Dunn J.R. (2015). The Value of Frameworks as Knowledge Translation Mechanisms to Guide Community Participation Practice in Ontario CHCs. Soc. Sci. Med..

[44] Arnstein S.R. (1969). A Ladder of Citizen Participation. J. Am. Inst. Plan..

[45] Cornwall A. (2008). Unpacking “Participation”: Models, Meanings and Practices. Community Dev. J..

[46] Public Health England (2015). NHS England. A Guide to Community-Centred Approaches for Health and Wellbeing. Full Report.

[47] Montesanti S.R., Abelson J., Lavis J.N., Dunn J.R. (2017). Enabling the Participation of Marginalized Populations: Case Studies from a Health Service Organization in Ontario, Canada. Health Promot. Int..

[48] Labonte R.N. (1993). Health Promotion and Empowerment: Practice Frameworks.

